# Fluoride toxicity and mitigation strategies in acidophilic bioleaching microorganisms

**DOI:** 10.1007/s00253-025-13677-x

**Published:** 2026-01-23

**Authors:** Mareike Thea Fritze, Sabrina Hedrich

**Affiliations:** Department of Biosciences, TU Bergakademie Freiberg, Leipziger Str. 29, 09599 Freiberg, Germany

**Keywords:** Fluoride inhibition, Acidophilic bacteria, Bioleaching, Fluoride complexation

## Abstract

**Abstract:**

Bioleaching is an established process for sulfidic ores and is increasingly applied to the recycling of industrial residues. However, unlike ores, many residues like sludge contain inhibitory elements, among which fluoride poses a major challenge due to its toxicity toward acidophilic microorganisms even at low concentrations. This study systematically investigated fluoride tolerance in pure and mixed cultures of various acidophilic sulfur- and iron-oxidizing bacteria commonly used for bioleaching, including *Acidithiobacillus* spp., *Leptospirillum* spp., and *Sulfobacillus thermosulfidooxidans*. Fluoride toxicity was found to be substrate-dependent. During sulfur oxidation, *A. thiooxidans* displayed the highest fluoride tolerance (0.5 mM F⁻), whereas *S. thermosulfidooxidans* showed complete inhibition. In contrast, iron-oxidizing bacteria demonstrated increased fluoride tolerance, with *S. thermosulfidooxidans* remaining active at 1.5 mM F⁻ when grown on ferrous iron. Mixed cultures showed enhanced fluoride tolerance during sulfur oxidation but reduced tolerance during iron oxidation. pH was identified as a critical factor influencing fluoride toxicity due to increased formation of undissociated HF at low pH. To mitigate fluoride inhibition, fluoride complexation with ferric iron or aluminum was evaluated. For *A. ferrooxidans*, iron oxidation resumed at Fe^3^⁺:F⁻ ratios of 7.5:1, while other cultures required ratios of at least 10:1. Aluminum complexation required Al:F⁻ ratios between 1:1 and 2:1, depending on the culture and growth conditions. Overall, fluoride inhibition during bioleaching is influenced by multiple factors, including pH, ferric iron concentration, and the fluoride dissolution rate. Early addition of aluminum is recommended to prevent microbial inhibition and ensure stable bioleaching performance.

**Key points:**

• *Higher fluoride tolerance was observed during iron oxidation.*

• *S. thermosulfidooxidans remained active up to 1.5 mM F⁻.*

• *Fluoride toxicity is strongly pH dependent due to increased HF formation at low pH.*

• *Effective fluoride complexation requires higher Fe*^*3+*^*:F⁻ ratios (> 7.5:1) than Al3⁺:F⁻ ratios (> 1:1)*

**Graphical Abstract:**

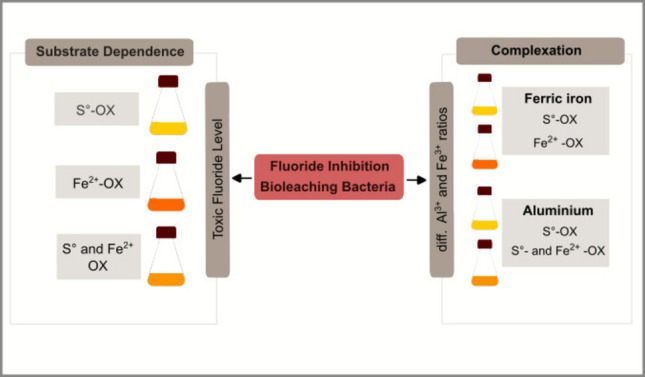

**Supplementary Information:**

The online version contains supplementary material available at 10.1007/s00253-025-13677-x.

## Introduction

Bioleaching, a process well established for the extraction of metals from ores using microorganisms (Vera et al. 2022), is also a promising approach for the recycling of metals from industrial residues and wastes (Kinnunen & Hedrich 2023). Acidophilic bacteria and archaea are most commonly applied in bioleaching (Vera et al. 2022). They grow optimally at pH below 3.0, are mostly chemolithoautotrophic, and obtain energy by oxidizing iron and reduced inorganic sulfur compounds (RISCs). The resulting ferric iron and sulfuric acid can serve as lixiviant for metal leaching from ore or waste material (Vera et al. 2022; Kinnunen & Hedrich 2023). Typical bioleaching microorganisms are, for example, the iron and/or sulfur-metabolizing *Acidithiobacillus* (*At*.) spp., the iron-oxidizing *Leptospirillum* (*L*.) spp., or moderately thermophilic Firmicutes (*Alicyclobacillus* spp*., Sulfobacillus* (*S*.) spp. (Vera et al. 2022). However, compared to ore bioleaching, the bioleaching of waste and residues causes several challenges (Gaustad et al. 2021). They are highly complex in their composition and may contain high concentrations of toxic compounds, including metals, salts, organic compounds (e.g., from plastics, resins), chloride, or fluoride (Kinnunen & Hedrich 2023).

Fluoride inhibition during bioleaching, primarily caused by the dissolution of fluoride from ores, has been widely reported (Dopson et al. 2008; Brierley & Kuhn 2010; Sicupira et al. 2011; Rodrigues et al. 2016, 2019; Reichel et al. 2017). It also poses a significant challenge for bioleaching of secondary materials, such as slags (Gahan et al. 2009; Han et al. 2012; Yang et al. 2020), sludges (Liu et al. 2011; Fritze & Hedrich 2024a), or black mass (Golmohammadzadeh et al. 2022; Fritze & Hedrich 2024b). These findings highlight the importance of understanding the effects of fluoride on microbial activity to optimize bioleaching processes for secondary materials.

While acidophiles have been reported to be highly tolerant to sulfate and most metal cations, they are very sensitive to anions, including chloride, fluoride, and small organic acids (Dopson & Holmes 2014; Falagán & Johnson 2018). Their enhanced metal resistance and sensitivity towards anions are most likely related to their internal positive membrane potential, which is caused by the intracellular accumulation of inorganic cations such as potassium (Dopson & Holmes 2014; Falagán & Johnson 2018). The positive charge ensures inherent resistance to cationic metals and facilitates the influx of permeable anions to maintain a near-neutral pH within the cytoplasm. However, an influx of toxic anions such as chloride or fluoride into the cells will disrupt the membrane potential and lead to a general intoxication/acidification of the cytoplasm (Norris & Ingledew 1992; Falagán & Johnson 2018).

**Fig. 1:**
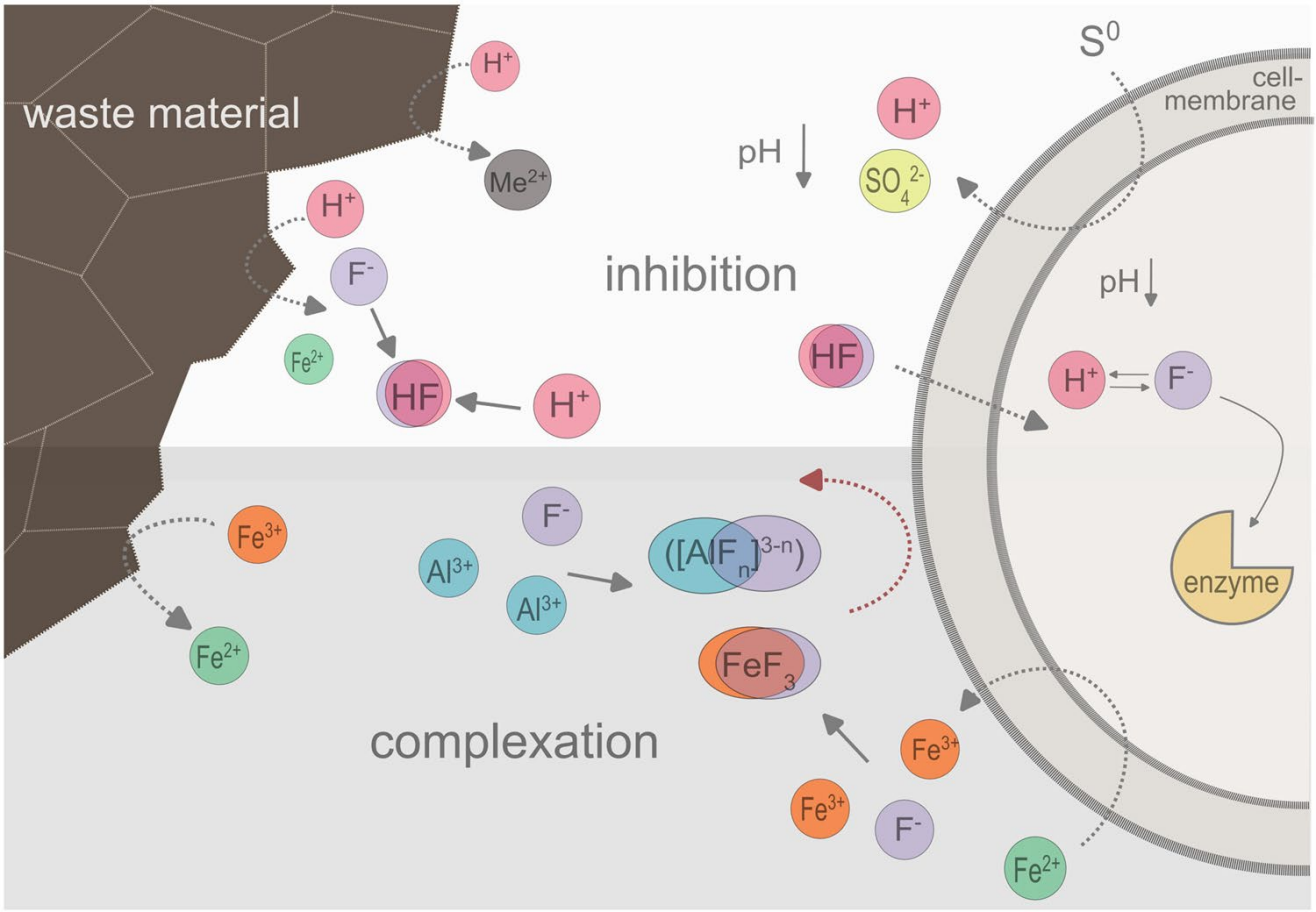
Mechanism of fluoride inhibition in acidophilic prokaryotes during bioleaching and complexation of the fluoride by aluminum and ferric iron ions.

The inhibition of microbial activity by fluoride is pH dependent, whereby it has no inhibitory effect at pH ≥ 7.0, is weak at pH 4.5, and strong at pH < 2.3 (Suzuki et al. 1999; Brierley & Kuhn 2010). Figure 1 shows the mechanism of fluoride inhibition in acidophilic prokaryotes during bioleaching. At low pH, fluoride mainly occurs as the undissociated free acid HF, which can easily enter the microbial cells (Suzuki et al. 1999; Brierley & Kuhn 2010). The permeability of HF through the lipid bilayer of the cell membrane is seven times higher than that of the fluoride anion (F^−^) (Gutknecht & Walter 1981). Thus, HF can pass through the cell membrane and dissociate into H^+^ and F^−^ inside the cell. The protons lead to a decrease of the cytoplasmic pH, while the fluoride anions can bind to some enzymes and reduce metabolic activity (Sircupia et al. 2011; Veloso et al. 2012). It is assumed that the decrease in the internal cell pH (caused by HF diffusion) forces the system to pump out protons to compensate for the diffusion of HF molecules into the cell. Overall, the energy requirement is increased, which leads to enhanced substrate consumption without increasing the biomass yield (Veloso et al. 2012). Ultimately, both the cytoplasmic acidification and the enzymatic inhibition caused by fluoride can result in cell death (Sircupia et al. 2011; Veloso et al. 2012).

However, in extreme environments, microorganisms are generally equipped with complex metabolic pathways to adapt to certain stress situations (Dopson & Holmes 2014). The fluoride tolerance mechanism is based on the regulation of the cell membrane and detoxification metabolism (Suzuki et al. 1999; Brierley & Kuhn 2010; Qian et al. 2013). In *At. ferrooxidans*, resistance mechanisms including maintaining membrane fluidity and permeability through modified biosynthesis of membrane components, adjusting the ratio of unsaturated to saturated fatty acids, and activating iron- and sulfur-metabolic pathways to supply energy (Ma et al. 2016). Nitrogen and phosphorus metabolism, as well as stress response transporters and regulatory systems, are also upregulated to limit protein and nucleotide damage (Ma et al. 2016).

During leaching of fluoride-containing waste material, fluoride is released. Due to the low pH during bioleaching, fluoride is mainly present as HF, which can easily enter the cell membrane. Within the cytoplasm, HF dissociates to H^+^ and F^−^, whereby the protons lead to a decrease of the neutral cytoplasm pH, while F^−^ can bind to some enzymes and reduce metabolic activity. Fluoride inhibition can be counteracted by complexation with aluminum or ferric iron. The HF concentration is reduced by complexing the fluoride. In addition, the complexes cannot diffuse into the cell.

Razzell and Trussel (1963) firstly studied fluoride inhibition in *At. ferrooxidans*, whereby a sodium fluoride concentration of 0.4 mM caused 30% inhibition and a concentration of 1.6 mM resulted in complete inhibition of microbial iron oxidation. Ma et al. (2017) described that the fluoride tolerance of *At. ferrooxidans* varies from 0.1 to 10 mM, depending on the substrate and growth stage, which has also been reported in other studies (Brierley & Kuhn 2010; Sicupira et al. 2011; Ma et al. 2017). Iron-grown *At. ferrooxidans* exhibit a higher fluoride tolerance than sulfur-grown cultures. Moreover, fluoride exhibited a stronger inhibitory effect when introduced prior to the log phase, which could be explained by the fact that cell adsorption to a solid substrate such as S° is required for utilization (Ma et al. 2017). However, the toxic effect of fluoride could have a detrimental effect on the cell membrane and thus lead to a reduction in microbial adsorption capacity. The use of iron as a substrate is not affected by the cell adsorption capacity (Ma et al. 2017). Furthermore, the literature indicates that fluoride resistance varies between species. Ma et al. (2013) studied the fluoride tolerance of five bioleaching strains, finding that *At. ferrooxidans* had the highest tolerance, followed by *At. thiooxidans*, *L. ferriphilum*, and *At. caldus*. *S. thermosulfidooxidans* showed the lowest tolerance. However, fluoride was added only during the exponential growth phase, and growth was monitored over a short period. Studies on other toxic metal cations and anions have shown a species-dependent inhibition (e.g., Rawlings 2005; Cabrera et al. 2005; Mangold et al. 2013; Falagán & Johnson 2018). Falagán & Johnson (2018) showed that the tolerance of various acidophilic species to copper and chloride varied greatly depending on different substrates supplied.

Various options to overcome fluoride inhibition have been reported, such as adaptation of the microorganisms to fluoride (Wang & Qiu 2011; Qian et al. 2013; Zhou et al. 2019). Wang & Qiu (2011) reported that microorganisms were able to grow in the presence of 45 mM F^−^ after continuous adaptation. The toxicity of HF can also be reduced by complexation or precipitation reactions (see Fig. 1). As a small ion with low polarizability in aqueous solution, fluoride forms relatively stable complexes with transition metal cations (Sicupira et al. 2011; Rodrigues et al. 2015; Ma et al. 2019). Various authors suggested that fluoride toxicity can be overcome by adding aluminum to the system (Brierley & Kuhn 2010, Sircupia et al. 2011; Veloso et al. 2012). Aluminum forms a strong complex with fluoride (see Eq. 1–4) (Goldstein 1964; Veloso et al. 2012), preventing the diffusion of fluoride into the bacterial cell. The complexation reduces the concentration of free fluoride ions and HF (Brierley & Kuhn 2010).

1 $$\mathrm A1^{3+}+\mathrm F^-\;\leftrightarrows\;\mathrm A1\mathrm F^{2+}$$

2 $$\mathrm A1^{3+}+2\mathrm F^-\;\leftrightarrows\;\mathrm A1\mathrm F_2^+$$

3 $$\mathrm A1^{3+}+3\mathrm F^-\;\leftrightarrows\;\mathrm A1{\mathrm F}_{3\left(\mathrm{aq}\right)}$$

4 $$\mathrm A1^{3+}+4\mathrm F^-\;\leftrightarrows\;\mathrm A1\mathrm F_4^-$$

Veloso et al. (2012) studied the influence of fluoride on the iron oxidation kinetics in *S. thermosulfidooxidans*. The fluoride toxicity was minimized at an Al/F molar ratio of 2:1, enabling copper bioleaching (Veloso et al. 2012), whereby Sicupira et al. (2011) showed that bioleaching was feasible with a minimum Al/F ratio of 1.4. In addition, the positive effect of ferric iron on fluoride complexation has also been described in the literature (Rodrigues et al. 2016, 2019; Ma et al. 2017). Equations (5–7) (Connick et al. 1956) present the respective complexation reactions:

5 $$\mathrm{Fe}^{3+}+\mathrm{HF}^-\;\leftrightarrows\;\mathrm{FeF}^{2+}+\mathrm H^+$$

6 $$\mathrm{Fe}F^{2+}+\mathrm{HF}^-\;\leftrightarrows\;\mathrm{FeF}_2^++\mathrm H^+$$

7 $$\mathrm{Fe}F_2^++\mathrm{HF}^-\;\leftrightarrows\;{\mathrm{FeF}}_3+\mathrm H^+$$

The critical fluoride concentration for *At. ferrooxidans* was below 0.1 mM when grown on sulfur, but increased to 1.0 mM when cultivated on iron (Ma et al. 2017). When microorganisms oxidize ferrous iron, the resulting ferric iron can form complexes with fluoride (e.g., FeF^2^⁺), thereby reducing the concentration of HF in the medium (Rodrigues et al., 2015, 2019; Ma et al. 2017). Further investigations showed that at a Fe:F ratio of 10:1, the concentration of HF was less than 2% of the total fluoride concentration (Ma et al. 2017).

Fluoride inhibition may also be reduced by the presence of jarosite, a ferric iron sulfate mineral (KFe_3_(SO_4_)_2_(OH)_6_), a s the effective ionic radii (r) of hydroxide and fluoride ions for tetrahedral coordination are very similar (r(OH^−^) = 0.121 nm and r(F^−^) = 0.117 nm, (Shannon 1976)), which could allow an exchange between the ions (Gunneriusson et al. 2009). Gunneriusson et al. (2009) studied the ability of jarosite to trap fluoride during precipitation and showed that fluoride was sorbed onto jarosite and a structural incorporation of fluoride occurred depending on the pH and fluoride concentration. When applied for bioleaching, the proportion of fluoride inclusions in the jarosite proved to be low, which means that it is not a feasible application for bioleaching.

Based on previous studies by the authors, demonstrating that fluoride inhibits microbial activity during the bioleaching of metal hydroxide sludge (Fritze & Hedrich 2024a) and LFP black mass (Fritze & Hedrich 2024b), fluoride inhibition of typical acidophilic bioleaching bacteria required more detailed studies. The aim of this study is to investigate the fluoride tolerance for the respective microorganisms of various *Acidithiobacillus* ssp., *Leptospirillum* ssp., and *S. thermosulfidooxidans* in pure and mixed cultures, dependent on the substrate supplied. An enrichment culture from acid mine water (dominated by sulfur oxidizers) with remarkable fluoride tolerances is also studied. To overcome fluoride inhibition during bioleaching, various options to complex fluoride, such as adding defined concentrations of aluminum and/or ferric iron, are investigated to determine the necessary ratios. The aim of this study is to obtain an overview of the fluoride inhibition of the bioleaching bacteria used in the studies of Fritze & Hedrich (2024a, b) and determine strategies to overcome the fluoride toxicity during bioleaching of fluoride-containing sludge and LFP black mass.

## Experimental

### Cultures and cultivation conditions

Typical mesophilic and moderately thermophilic iron- and/or sulfur-oxidizing acidophilic autotrophic bacteria applied in bioleaching were used in this study (Table 1). All strains were obtained from the German Collection of Microorganisms and Cell Cultures (DSMZ, Braunschweig, Germany).

**Table 1:** Overview of selected microorganisms, their metabolism and optimum growth conditions (S° = sulfur oxidation, Fe^2+^ = iron oxidation)

Strain	S°	Fe^2+^	pH	T [°C]	Refs.
*Acidithiobacillus *(*At*.)* ferrooxidans*^T^	x	x	2.5	30–35	(Kelly & Wood 2000)
*At. thiooxidans*^T^	x		2.0–3.0	28–30	(Kelly & Wood 2000)
*At. caldus*^T^	x		2.0–2.5	45	(Kelly & Wood 2000)
*Leptospirillum *(*L.*) *ferriphilum*^T^		x	1.4–1.8	30–37	(Coram and Rawlings 2002)
*L. ferrooxidans* DSM 2391		x	2.5–3.0	30	(Hippe 2000)
*Sulfobacillus *(*S.*) *thermosulfidooxidans*^T*^	x	x	3.0	45	(Zhang et al. 2021)

*Mixotrophic

For cultivation experiments, the basal salt medium (HBS) (Nancucheo et al. 2016) was used, with 1 l of medium consisting of 20 ml solution A (50×) containing in gram per liter: Na_2_SO_4_∙10H_2_O 7.5; (NH_4_)_2_SO_4_ 22.5; KCl 2.5; MgSO_4_∙7H_2_O 25; KH_2_PO_4_ 2.5; Ca(NO_3_)_2_·4H_2_O 0.7, supplemented with 1 ml 1000 × trace element solution containing in gram per liter: ZnSO_4_·7 H_2_O 10; CuSO_4_·5 H_2_O 1.0; MnSO_4_·4 H_2_O 1.0; CoSO_4_·7H_2_O 1.0; Cr_2_(SO_4_)_3_·15 H_2_O 0.5; H_3_BO_3_ 0.6; Na_2_MoO_4_·2H_2_O 0.5; NiSO_4_·6 H_2_O 1.0; Na_2_SeO_4_·10 H_2_O 1.0; Na_2_WO_4_·2 H_2_O 0.1, added up to 1 l with distilled water and pH adjusted to 1.8 with sulfuric acid. Cultivation was carried out in a volume of 25 ml in 100-ml shake flasks at 120 rpm. The cultivation time for the pre-cultures was 7–10 days until microbial activity was clearly observed. The inoculum used was 10% (w/v) for all experiments. For that, the (suspended) cell count was determined using a Thoma counting chamber (0.100 mm × 0.0025 mm^2^; Marienfeld, Germany).

The acidophiles were cultivated in pure culture and in two mixed cultures (see Table 2 for the conditions). Depending on the metabolism, cultivation was carried out on sulfur or iron as a substrate. Bacteria that were able to metabolize both were cultivated separately on each substrate. The tested mixed cultures include the mesophiles *At. ferrooxidans* and *L. ferrooxidans* (LA), and the moderate thermophiles *S. thermosulfidooxidans*, *L. ferriphilum*, and *At. caldu*s. These were both cultivated using S° and Fe^2+^ as substrates. The elemental sulfur (S°) was tyndalized at 112 °C beforehand. Ferrous iron was supplied from a 1 M stock of FeSO_4_ × 7H_2_O (pH 1.5). Since *S. thermosulfidooxidans* is mixotrophic, 0.02% yeast extract was added when it was cultivated in a pure culture.

**Table 2:** Overview of the pure and mixed cultures and cultivation conditions used in this study

	Strain/culture	Substrate	T [°C]
*Pure cultures*	*At. ferrooxidans*	1% sulfur	30
	*At. thiooxidans*	1% sulfur	30
	*At. caldus*	1% sulfur	42
	*S. thermosulfidooxidans*	1% sulfur, 0.02% YE	42
	*L. ferriphilum*	50 mM Fe^2+^	42
	*L. ferrooxidans*	50 mM Fe^2+^	30
	*At. ferrooxidans*	50 mM Fe^2+^	30
	*S. thermosulfidooxidans*	50 mM Fe^2+^, 0.02% YE	42
*Mixed cultures*	*S. thermosulfidooxidans*, *L. ferriphilum *&* A. caldus*	1% sulfur 50 mM Fe^2+^	42
	*L. ferrooxidans *&* At. ferrooxidans*	1% sulfur 50 mM Fe^2+^	30

### Fluoride inhibition tests based on substrates

For the inhibition studies, sodium fluoride (NaF) was prepared as a 100 mM stock solution with distilled water, pH 1.8. For the test with sulfur as substrate, *At. thiooxidans*, *At. ferrooxidans*, *At. caldus*, and *S. thermosulfidooxidans* were used. The cultures were cultivated as shown in Table 2 with the addition of the corresponding fluoride concentration. Pre-cultures were used, which were grown on the respective substrate. The following fluoride concentrations were tested for the sulfur substrate: 0.25 mM, 0.5 mM, and 0.75 mM. For the test with iron as substrate, *At. ferrooxidans*, *L. ferrooxidans*, *L. ferriphilum*, and *S. thermosulfidooxidans* were used, whereby the following fluoride concentrations were investigated: 0.5 mM, 1 mM, and 1.5 mM. For the test with the mixed cultures, both sulfur and iron as substrates, the following fluoride concentrations were tested: 0 mM, 0.5 mM, 1 mM, and 1.5 mM. The experiments with the different fluoride concentrations were carried out in triplicates. A positive control without fluoride was also set up. A chemical control (uninoculated) containing the highest concentration of fluoride (1.5 mM) was included in each experiment. The cultivation was carried out for seven days, during which the pH (Mettler Toledo, InLab® Semi-Micro) and/or redox potential (Mettler Toledo, InLab® Redox Micro, vs. Ag/AgCl electrode) were measured regularly. In addition, the ferrous, ferric, and total iron concentrations were determined for the cultures containing iron by using the Ferrozine® assay (Lovley & Phillips 1987; Pascualreguera et al. 1997).

### Fluoride inhibition in enrichment culture

In order to enrich acidophilic sulfur- and iron-oxidizing or -reducing acidophiles for the bioleaching of metal hydroxide sludge (Fritze & Hedrich 2024a), enrichment cultures were set up in the presence of S°, ferrous iron, and sludge containing ferric iron, gallium, arsenic, and fluoride. The enrichment cultures were inoculated with sludge and water samples from the abandoned Reiche Zeche silver mine in Freiberg (Saxony). One aerobic enrichment culture showed promising microbial activity in the presence of up to 3% (w/v) metal hydroxide sludge (HBS media pH 1.8, 1% (w/v) S° as substrate). The culture showed active sulfur oxidation and was dominated by *Acidithiobacillus* sp. as confirmed by molecular fingerprinting T-RFLP (Terminal Restriction Fragment Length Polymorphism) analysis (Hedrich et al. 2016). The culture was examined for its fluoride tolerance with sulfur as substrate in combination with ferric iron, adjusted to a final concentration of 40 mM, accounting for the ferric iron already present in the sludge. Cultivation was performed in HBS media, pH 1.8, at 30 °C cultivation temperature. The maximum concentration released from 3% (w/v) metal hydroxide sludge was calculated to be 8.5 mM F. Fluoride was used as sodium fluoride with the following concentrations: 0 mM, 4 mM, 8 mM, and 12 mM. All experiments were carried out in triplicates. Cultivation was conducted for seven days, during which the pH and/or the redox potential were measured. In addition, the ferrous, ferric, and total iron concentrations were determined for the cultures containing iron.

### Fluoride complexation with ferric iron

As ferric iron also forms complexes with fluoride and can reduce the inhibition of microbial activity by HF, experiments investigating the addition of different Fe^3+^:F ratios were performed. Fluoride was added as NaF and ferric iron as (Fe_2_(SO_4_)_3_ × xH_2_O). Experiments were carried out with pure cultures using either 1% sulfur (*At. thiooxidans* and *At. ferrooxidans*) or 50 mM Fe^2+^ as substrate (*At. ferrooxidans* and *L. ferrooxidans*). For inoculation, pre-cultures were used, which were grown on the respective substrate. The following Fe^3+^:F ratios were tested based on preliminary experiments: 0:1, 2.5:1, 5:1, 7.5:1, 10:1, 15:1. A concentration of 3 mM F was set in the medium. All experiments were carried out in triplicates (except for the 0:1 ratio). A positive control with no addition of fluoride and a chemical control (uninoculated) with the highest concentration of fluoride and iron added in each experiment were included. Cultivation was conducted for seven days, during which the pH and/or the redox potential were monitored. In addition, the ferrous, ferric, and total iron concentrations were determined.

### Fluoride complexation with aluminum

Aluminum can also be used to complex fluoride to prevent microbial inhibition. For this purpose, various Al:F ratios were tested with cultures used in the respective bioleaching studies by Fritze & Hedrich (2024a, b). Aluminum sulfate (Al_2_(SO_4_)_3_ × xH_2_O, pH 1.8) and sodium fluoride were used. A concentration of 2.5 mM F was set in the medium. The investigations were carried out with *At. thiooxidans*, using 1% sulfur alone, and 1% sulfur with 30 mM ferric iron as substrates. Ferric iron was added because bioleaching with *At. thiooxidans* was to be conducted on a residue containing ferric iron. The concentration was calculated based on the expected ferric iron levels under practical conditions. For both conditions, the following Al:F ratios were tested: 0:1, 1:1, 1.25:1, and 1.5:1. Also, a mixed culture of the moderately thermophilic organisms *S. thermosulfidooxidans*, *L. ferriphilum*, and *At. caldus* was tested with 1% sulfur and 50 mM Fe^2^⁺ as the substrate, using the following Al:F ratios: 1.5:1, 2:1, and 2.5:1.

Experiments were performed in triplicates (except for the 0:1 ratio). In addition, a positive control (without Al and F) and a chemical control (uninoculated) with the highest concentration of fluoride added in each experiment were also carried out. The cultivation time was seven to ten days, during which the pH, the redox potential, and the iron concentrations were monitored.

## Results

### Substrate-dependent fluoride inhibition

#### Fluoride inhibition in pure cultures

The effect of fluoride on the activity of various acidophilic iron- and sulfur-oxidizing bacteria was investigated. The cell counts of the inoculum for the growth experiments using S^0^ as the substrate were 1.62 × 10^8^ cells/ml for *At. thiooxidans*, 6.4 × 10^7^ cells/ml for *At. ferrooxidans*, 1.74 × 10^8^ cells/ml for *At. caldus*, and 3.52 × 10^7^ cells/ml for *S. thermosulfidooxidans*.

**Fig. 2:**
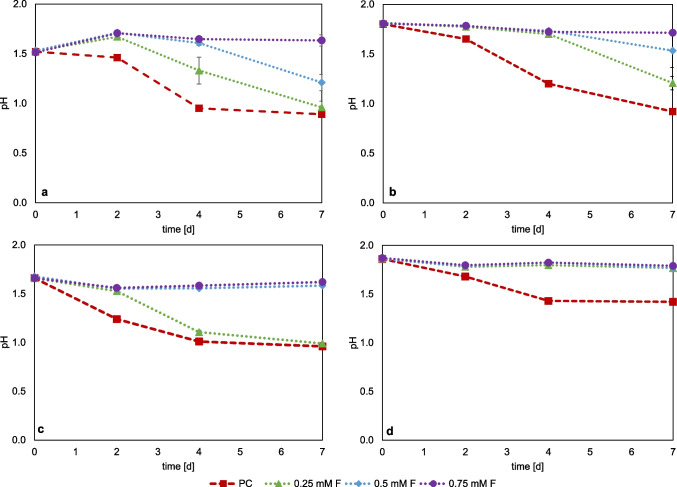
Monitoring of pH value in pure cultures of sulfur-oxidizing bacteria at different fluoride concentrations; *At. thiooxidans* (**a**), *At. ferrooxidans* (**b**), *At. caldus* (**c**), *S. thermosulfidooxidans* + 0.02% yeast extract (**d**); PC = positive control without addition of fluoride. (Data represent mean values of triplicate set ups with standard deviation)

Figure 2 a shows the monitoring of pH in cultures of *At. thiooxidans* with sulfur. The positive control (PC), without the addition of fluoride, shows a clear drop in pH to 0.97 by day seven. The pH decrease confirms the active microbial sulfur oxidation, which leads to the formation of sulfuric acid. With the addition of fluoride, a delayed pH decrease occurs with increasing concentration of fluoride within the 7-day cultivation period. Addition of 0.25 mM F led to a delay in the pH drop until day two. This shows that this concentration has only a low inhibiting effect on the sulfur oxidation activity of *At. thiooxidans*. With the addition of 0.5 mM F, this delay was observed until day four, with a drop to pH 1.21 only on day seven, showing that inhibition does not occur immediately but causes a “lag effect” for sulfur oxidation. Addition of 0.75 mM F resulted in complete inhibition of the sulfur oxidation activity. In the experiments with *At. ferrooxidans* (Fig. 2b), only a slight drop to pH ~ 1.70 was observed in all set ups with fluoride until day four. The continued decline in pH was less pronounced at higher fluoride concentrations. On day seven, the pH for 0.25 mM F addition was pH 1.21, for 0.5 mM F pH 1.53, and for 0.75 mM F addition the pH remained at 1.7. It can therefore be inferred that sulfur oxidation by *At. ferrooxidans* was strongly inhibited at 0.75 mM F. For *At. caldus* (Fig. 2c), the addition of 0.25 mM F initially showed a delay in the drop of pH and a lag effect due to fluoride stress. For 0.5 mM F and 0.75 mM F additions, no noticeable pH drop was observed by day seven, suggesting a stronger inhibition of the sulfur oxidation activity. For *S. thermosulfidooxidans* (Fig. 2d), only the positive control showed a drop in pH from 1.80 to 1.42. For the approaches with fluoride, there was no drop in pH, indicating complete inhibition of sulfur oxidation under these conditions. Of all the sulfur-grown cultures tested, *At. thiooxidans* showed the best activity even at fluoride concentrations of 0.5 mM F, while *At. ferrooxidans* showed reduced activity at this concentration. *At. caldus* showed no activity at concentrations above 0.25 mM F. For *S. thermosulfidooxidans*, it is concluded that all tested fluoride concentrations had a lethal effect on microbial sulfur oxidation.

**Fig. 3:**
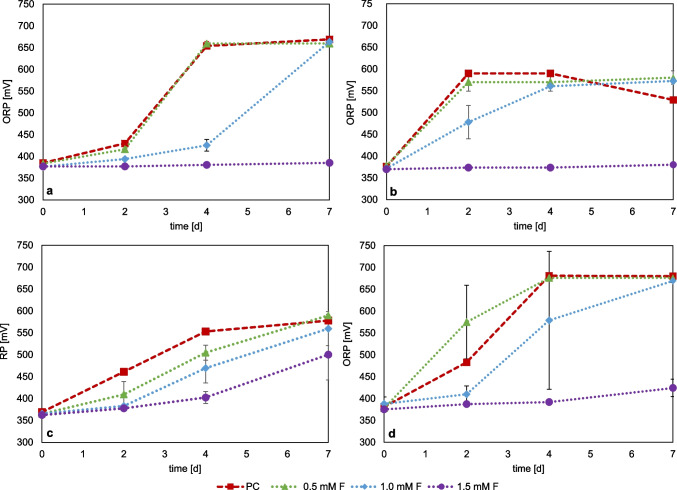
Monitoring of redox potential (vs. Ag/AgCl) in pure cultures of iron-oxidizing bacteria with different fluoride concentrations; *L. ferrooxidans* (**a**), *At. ferrooxidans* (**b**), *S. thermosulfidooxidans* + 0.02% yeast extract (**c**), and *L. ferriphilum* (**d**), PC = positive control without addition of fluoride. (Data represent mean values of triplicate set ups with standard deviation)

Figure 3 shows the redox potential monitoring of the fluoride inhibition tests for the iron-oxidizing species *L. ferrooxidans* (Fig. 3a), *At. ferrooxidans* (Fig. 3b), *S. thermosulfidooxidans* (Fig. 3c), and *L. ferriphilum* (Fig. 3d). The comparative ferrous iron concentration data are shown in Figure S1. The cell counts of the inoculum were 6.40 × 10^7^ cells/ml for *L. ferrooxidans*, 6.08 × 10^7^ cells/ml for *At. ferrooxidans*, 5.44 × 10^7^ cells/ml for *L. ferriphilum*, and 4.96 × 10^7^ cells/ml for *S. thermosulfidooxidans*.

*L. ferrooxidans* grew almost similar with 0.5 mM F as the positive control, with an increase in redox potential at day two from 420 to 660 mV, indicating that 0.5 mM F has no inhibitory effect. At 1 mM F, the increase of the redox potential was delayed until day 4. With the addition of 1.5 mM fluoride, no visible iron oxidation could be observed within the 7 days of cultivation. *At. ferrooxidans* behaved very similarly to *L. ferrooxidans*, with active iron oxidation at 0.5 mM F. The addition of 1.0 mM F showed less increase in the redox potential and thus a delay in iron oxidation activity until day four, while the addition of 1.5 mM showed no visible increase in redox potential during the experiment. Tests with *L. ferriphilum* showed similar trends, although fluctuations between the replicates occurred. The addition of 1.0 mM F also showed a delay in the increase in redox potential before reaching the same values as the positive control and the 0.5 mM F set up on day seven. At 1.5 mM F, only a slight increase in redox potential was observed from 375 to 420 mV within 7 days, indicating that *L. ferriphilum* already exhibited reduced iron oxidation activity at this concentration.

*S. thermosulfidooxidans* also showed a concentration-dependent delay of the iron oxidation activity with increasing fluoride concentrations, but at 1.5 mM F with the redox potential still increased from 360 to 500 mV within 7 days of incubation. Among the iron-grown cultures tested, *S. thermosulfidooxidans* exhibited the highest activity, maintaining iron oxidation even at 1.5 mM. F. *L. ferriphilum* also showed low activity at this concentration, whereas *At. ferrooxidans* and *L. ferrooxidans* showed activity only up to 1.0 mM.

#### Fluoride inhibition in mixed cultures

**Fig. 4:**
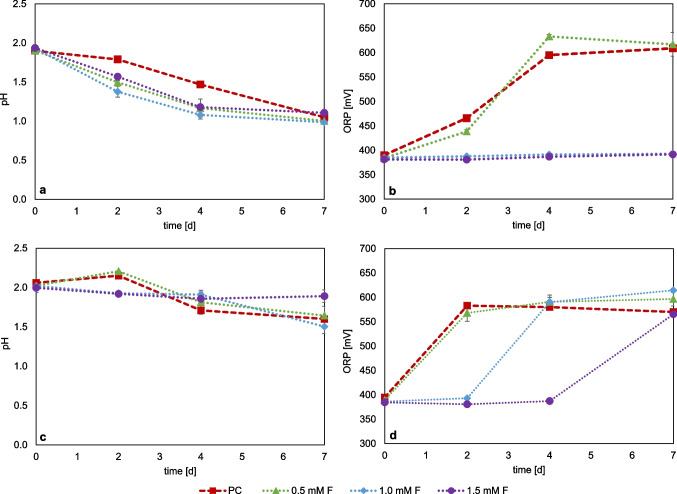
Monitoring of pH and redox potential (vs. Ag/AgCl) in mixed cultures of iron-and sulfur oxidizers under the addition of different fluoride concentrations; moderately thermophilic culture of *S. thermosulfidooxidans*, *L. ferriphilum*, and *At. caldus* (**a**, **b**); mesophilic culture of *L. ferrooxidans* and *At. ferrooxidans* (**c**, **d**), PC = positive control without the addition of fluoride. (Data represent mean values of triplicate set ups with standard deviation)

Bioleaching of primary and secondary resources can be enhanced by mixed microbial cultures (Kinnunen & Hedrich 2023; Hedrich et al. 2016). Based on studies of fluoride-containing industrial residues (Fritze & Hedrich 2024a, b), the fluoride tolerance of mesophilic (*At. ferrooxidans*, *L. ferrooxidans*) and moderately thermophilic (*S. thermosulfidooxidans, L. ferriphilum*, *At. caldus*) mixed cultures was investigated. The cell counts of the inoculum were 1.6 × 10^8^ cells/ml for the moderate thermophilic mixed culture (42 °C) and 1.82 × 10^8^ cells/ml for the mesophilic culture (30 °C). Figure 4 shows the monitoring of the pH and the redox potential of both mixed cultures with the addition of different fluoride concentrations. The comparative ferrous iron concentration is shown in Figure S2. A drop in pH was observed in the moderately thermophilic mixed culture for all three fluoride concentrations, which shows that active microbial sulfur oxidation occurred. Set ups with 1.5 mM F showed a pH of 1.1 after seven days of cultivation, and set ups with lower fluoride concentrations showed a pH of approx. 1.0. Thus, compared to the pure cultures of *At. caldus* and *S. thermosulfidooxidans*, the mixed culture displayed tolerance even at 1.5 mM F. With regard to microbial iron oxidation, activity was only observed up to 0.5 mM fluoride for the moderately thermophilic mixed culture, which was indicated by an increase in redox potential. At 1.0 and 1.5 mM F, no increase in redox potential was determined, which is unexpected based on the results obtained with the pure culture studies of *L. ferriphilum* and *S. thermosulfidooxidans*. In pure culture, *L. ferriphilum* showed activity at 1.0 mM F, and *S. thermosulfidooxidans* even still at 1.5 mM F. However, it cannot be ruled out that one of the bacteria was suppressed in the mixed culture, although the positive control exhibited iron and sulfur oxidation activity.

For the mesophilic mixed culture (LA), a drop to pH 1.6 was observed after seven days at 0.5 mM F and from pH 2.0 to 1.5 at 1.0 mM F, indicating active sulfur oxidation by *At. ferrooxidans*. Again, the positive effect of using a mixed culture for microbial sulfur oxidation and fluoride tolerance was demonstrated. While *At. ferrooxidans* showed no activity at 0.75 mM in pure culture with sulfur as a substrate, it was active even at 1.0 mM in mixed culture. At 1.5 mM F, only a slight drop from pH 2.0 to 1.9 was observed. The redox potential in this mixed culture increased for the 0.5 mM F set ups already up to day 2, while a slight delay was observed for the higher concentrations even at 1.5 mM F. This fluoride tolerance was higher compared to the tested pure cultures of *L. ferrooxidans* and *At. ferrooxidans*, where activity was only observed up to 1.0 mM F. Both bacterial strains are capable of oxidizing Fe^2^⁺, the inhibitory effect of fluoride is less pronounced in the mixed mesophilic culture compared to the pure culture. This observation contrasts with the results obtained from the moderately thermophilic culture, where fluoride inhibition was more evident.

#### Fluoride inhibition in a fluoride-adapted enrichment culture

An acidophilic enrichment culture cultivated in the presence of a fluoride-containing, metal-rich sludge, which also contains elevated concentrations of ferric iron (Fritze & Hedrich 2024a), indicated tolerance to elevated concentrations of fluoride. According to terminal restriction fragment length (T-RFLP) analysis, the culture consisted predominantly of *Acidithiobacillus *sp., showing sulfur oxidation activity and only slight iron reduction activity. As previous experiments have shown that a high concentration of iron appears to have a positive effect on fluoride tolerance, the culture was tested in the presence of sulfur and sulfur/ferric iron. The cell counts of the inoculum were 4.80 × 10^7^ cells/ml for the sulfur-grown enrichment culture.

**Fig. 5:**
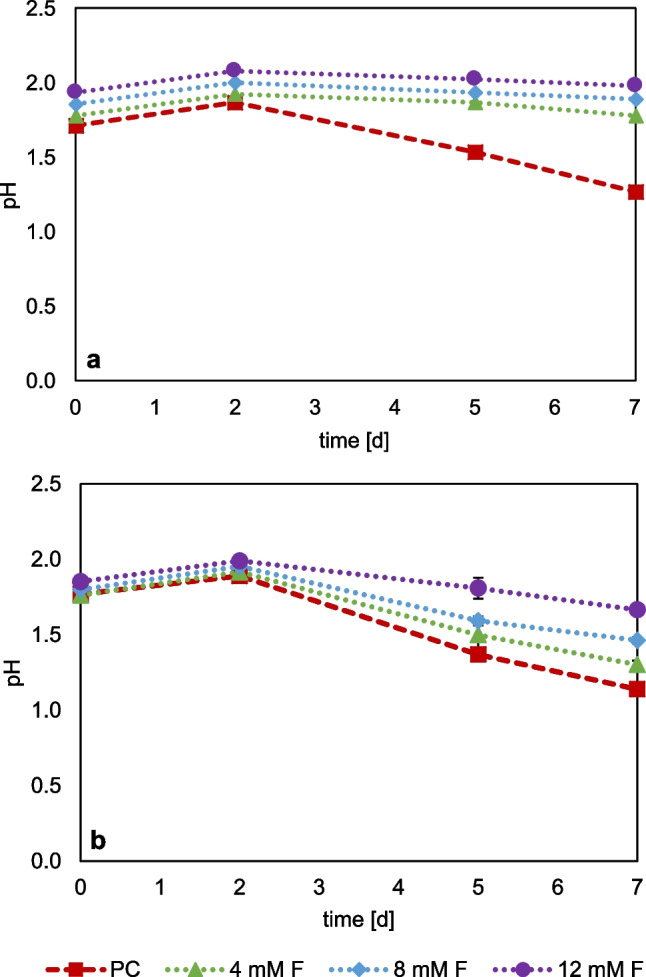
Monitoring of changes in pH of the enrichment culture at different fluoride concentrations when grown on sulfur (**a**) as well as on ferric iron and sulfur (**b**), PC = positive control without addition of fluoride. (Data represent mean values of triplicate set ups with standard deviation)

Figure 5 shows the development of the pH during cultivation on sulfur, as well as on sulfur and ferric iron. The comparative data of ferric iron concentration and redox potential is shown in Figure S3. By using sulfur as a substrate, there is only a slight drop in pH at 4 mM F compared to the set up without fluoride (positive control). For 8 and 12 mM, no notable drop in pH and thus no active sulfur oxidation was observed. When sulfur and ferric iron were added, a stronger drop in pH was observed at lower fluoride concentrations. Although, in contrast to sulfur-grown cultures, active sulfur oxidation occurred at 4 mM F. Activity decreased with increasing fluoride concentrations. The redox potential and the ferric iron concentration (see Appendix Figure S3) show a slight reduction of iron at 4 mM F. The high ferric iron concentrations enabled a higher fluoride tolerance of the acidophilic microorganisms in the enrichment culture, as ferric iron complexes HF.

### Complexation to mitigate fluoride inhibition

#### Complexation of fluoride using ferric iron

**Fig. 6:**
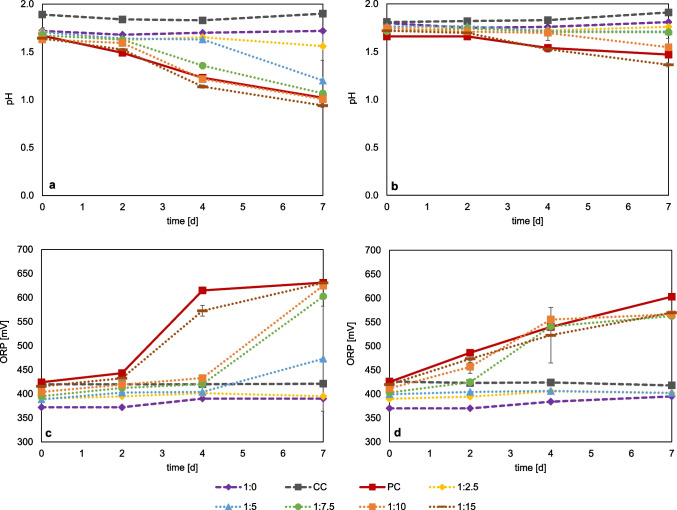
Monitoring of pH and redox potential (vs. Ag/AgCl) in pure cultures of iron- and sulfur-oxidizing bacteria at different molar ratios of fluoride and ferric iron (F:Fe^3+^), *At. thiooxidans* (**a**), *At. ferrooxidans* on S° (**b**), *L. ferrooxidans* (**c**), *At. ferrooxidans* on Fe^2+^ (**d**), CC = chemical control, PC = positive control without fluoride. (Data represent mean values of triplicate set ups with standard deviation)

As described in the literature, ferric iron can complex fluoride and thereby reduce the HF concentration (Ma et al. 2017, 2019). The ferric iron concentration required to reduce fluoride inhibition was determined in sulfur-grown *At. thiooxidans* and *At. ferrooxidans* cultures, as well as in iron-grown *At. ferrooxidans* and *L. ferrooxidans* cultures. The cell counts of the inoculum were 1.49 × 10^8^ cells/ml for *At. thiooxidans*, 6.88 × 10^7^ cells/ml for *At. ferrooxidans* (S°), 5.84 × 10^7^ cells/ml for *At. ferrooxidans* (Fe^2+^), and 6.00 × 10^7^ cells/ml for *L. ferrooxidans*. Figure 6 shows the monitoring of pH for *At. thiooxidans* and *At. ferrooxidans* with sulfur as substrate, as well as *At. ferrooxidans* and *L. ferrooxidans* with ferrous iron as substrate. The comparative ferrous iron concentration data are shown in Figure S4. For *At. thiooxidans* (Fig. 6a), the ratios 1:15 and 1:10 F:Fe^3+^ showed very similar behavior in the decrease of the pH compared to the positive control without fluoride, indicating that a ratio of 1:10 F:Fe^3+^ is sufficient to allow an active microbial sulfur oxidation. The pH dropped from approx. 1.7 to pH 1.0 within 7 days. At a F:Fe^3+^ ratio of 1:7.5, a slight delay in the drop of pH occurred until reaching a final pH of 1.07. For a ratio of 1:5.0, pH started to drop from 1.6 at day 4 to a final pH of 1.2. Adding ferric iron at a fivefold excess relative to fluoride supported microbial growth but did not completely prevent fluoride inhibition. At a F:Fe^3+^ ratio below 1:2.5, no pH and thus no microbial sulfur oxidation was observed for *At. thiooxidans*. *At. ferrooxidans* (Fig. 6b) displayed high microbial sulfur oxidation at 1:15 F:Fe^3+^ indicated by a pH decrease to 1.36 after 7 days. A delay in activity was observed for a ratio of 1:10 until day 4. For all other lower ratios tested, no microbial sulfur oxidation could be determined. *At. thiooxidans* showed sufficient activity at a F:Fe^3^⁺ ratio of 1:7.5, while *At. ferrooxidans* required at least 1:10 when grown on sulfur.

*At. ferrooxidans* was capable of oxidizing iron to a similar extent as the positive control at a F:Fe^3+^ ratio of 1:15 and 1:10 (Fig. 6c). The redox potential rose from approx. 420 mV to approx. 570 mV on day 7. For the 1:7.5 F:Fe^3+^ ratio, the redox potential only started rising from day 2 to a final redox potential of 570 mV. Although a slight delay in microbial iron oxidation was observed, Fe^2+^ was still oxidized, which had a positive effect on the inhibition by the HF ions. No iron oxidation activity was observed for all ratios < 1:7.5. The pH in cultures with a F:Fe^3+^ ratio > 1:7.5 was above 2.0 on day seven (data not shown), while for the lower ratios, the pH was around 1.9. Experiments with *L. ferrooxidans* showed that a ratio of 1:15 enabled high microbial iron oxidation. The redox potential increased to 630 mV after a slight delay. The ratios 1:10 and 1:7.5 showed a delay in iron oxidation, with a clear increase in redox potential occurring at day 4, rising to 630 and 602 mV, respectively, by day seven. No activity was observed for these iron oxidizers at the lower F:Fe ratios. The pH on day seven was between 2.1 and 2.2 (data not shown) in cultures with a F:Fe^3+^ ratio > 1:5.0.

*At. ferrooxidans* required a lower F:Fe^3^⁺ ratio than *L. ferrooxidans* to achieve effective iron oxidation. At a F:Fe^3^⁺ ratio of 1:7.5, *At. ferrooxidans* showed an increase in redox potential within two days, while *L. ferrooxidans* reached a similar redox potential only after four days. These results suggest that a F:Fe^3^⁺ ratio of approximately 1:10 may be sufficient to sustain iron oxidation activity in *L. ferrooxidans*.

#### Complexation of fluoride using aluminum

**Fig. 7:**
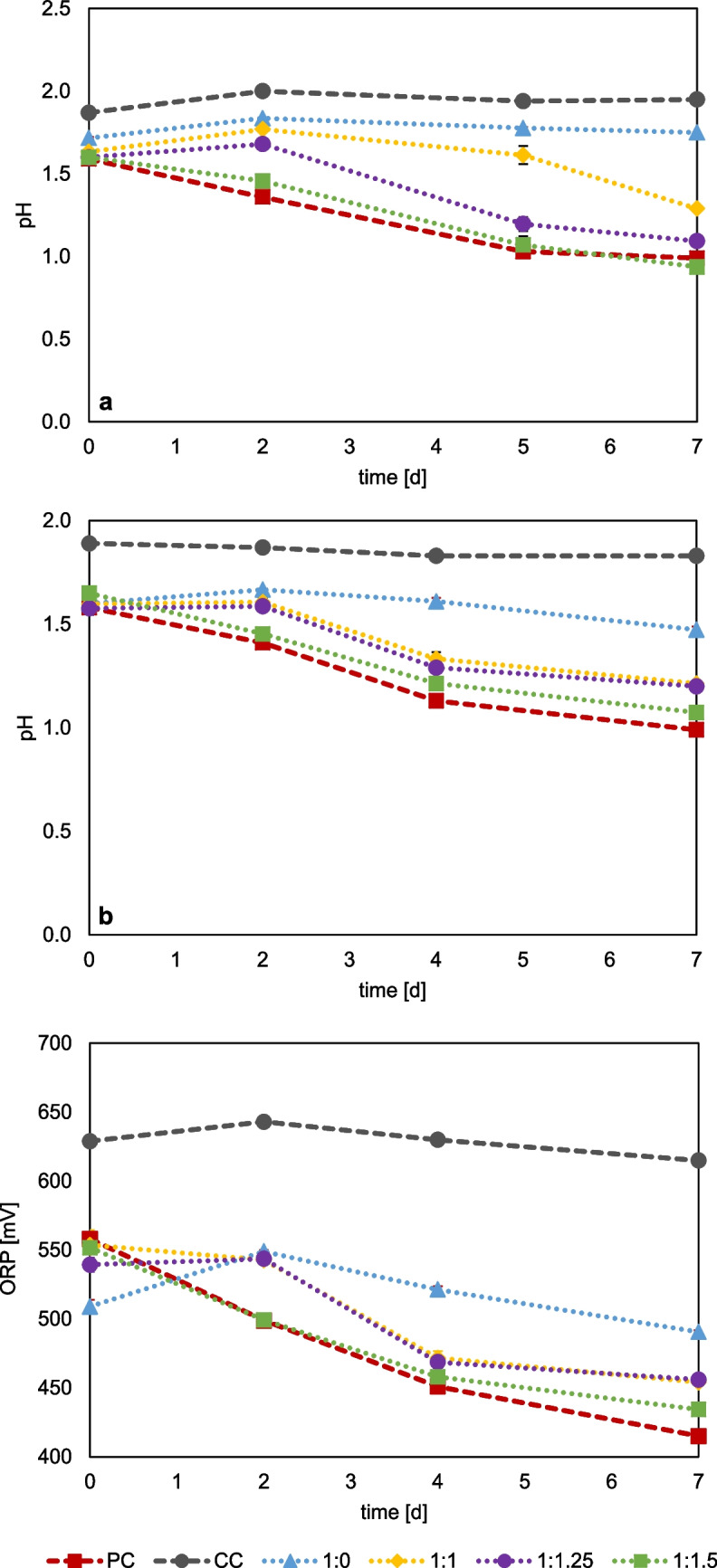
Monitoring of pH and redox potential (vs. Ag/AgCl) in pure cultures of *At. thiooxidans* at different molar ratios of aluminum and fluoride (Al:F). *At. thiooxidans* grown on 1% (w/v) sulfur (**a**), *At. thiooxidans* grown on sulfur 1% (w/v) and 30 mM ferric iron (**b**, **c**); CC = chemical control, PC = positive control without the addition of fluoride. (Data represent mean values of triplicate set ups with standard deviation)

Aluminum has previously been described as an effective complexing agent to overcome fluoride inhibition (Brierley & Kuhn 2010; Sicupira et al. 2011; Veloso et al. 2012). Therefore, studies with *At. thiooxidans* and a mixed culture of *S. thermosulfidooxidans*, *L. ferriphilum*, and *At. caldus* aimed to determine the optimum Al:F ratio for the cultivation. Initially, different Al:F ratios were tested for *At. thiooxidans* grown on sulfur as well as sulfur and ferric iron. The cell counts of the inoculum were 1.54 × 108 cells/ml for the moderate thermophilic mixed culture (42 °C) and 1.57 × 108 cells/ml for *At. thiooxidans*. When sulfur was used as substrate, high microbial activity was observed at an Al:F ratio of 1.25:1, evidenced by a fast decrease in pH (Fig. 7). At lower Al:F ratios, a slight delay in pH was observed from day five to seven, indicating that the Al concentration was too low to prevent fluoride inhibition. The pH progression at an Al:F ratio of 1.5:1 was very similar to that of the positive control (without the addition of aluminum and fluoride). Thus, fluoride complexation by aluminum was sufficient, and no inhibition of microbial sulfur oxidation was observed. Cultures additionally containing 30 mM ferric iron displayed a decrease in pH at an Al:F ratio of 1:1, indicating the positive effect of ferric iron, as lower Al:F ratios are sufficient to achieve high microbial activity in the presence of fluoride. Cultures containing 1.5:1 Al:F showed only minor differences in pH decrease compared to the positive control. Therefore, a ratio of at least 1.25:1 Al:F allows for high microbial activity of *At. thiooxidans* grown on sulfur. When ferric iron is present in the cultures, an Al:F ratio of at least 1:1 is sufficient. The comparative ferrous iron concentration data are shown in Figure S5.

**Fig. 8:**
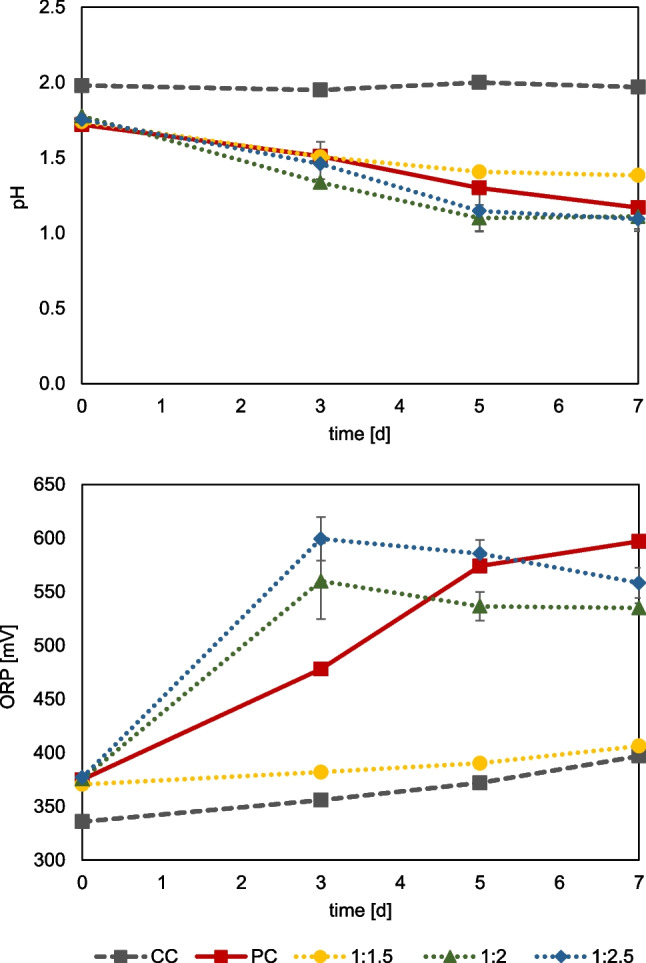
Monitoring of pH and redox potential (vs. Ag/AgCl) of a moderate thermophilic mixed culture (*S. thermosulfidooxidans*, *L. ferriphilum*, and *At. caldus*) grown on 1% sulfur (w/v) and 50 mM Fe^2+^ at different molar ratios of Al:F, CC = chemical control, PC = positive control without addition of fluoride. (Data represent mean values of triplicate set ups with standard deviation)

Different Al:F ratios were also tested for the moderate thermophilic mixed culture, based on preliminary tests. Figure 8 shows that at a ratio of 2:1 Al:F, a clear drop in pH from 1.75 to approx. 1.1 occurred after 7 days. At an Al:F ratio of 2.5:1, the decrease of pH to 1.1 was slightly stronger, while at 1.5:1 Al:F, the pH only decreases to 1.38. Microbial iron oxidation seemed completely inhibited at 1.5:1 Al:F, indicated by only minor change in redox potential. For the Al:F ratios of 2:1 and 2.5:1, an increase in redox potential is observed up to day three. Cultures with an Al:F ratio of 2.5:1 showed the highest redox potential with a mean of 599 mV, which fell slightly to 558 by day seven. The decrease may be due to ferric iron precipitation or the presence of reducing sulfur metabolites formed by *At. caldus* (Breuker & Schippers 2024). The data show that a ratio of at least 2:1 Al:F is required to avoid fluoride inhibition of this mixed culture under these conditions. The relative ferrous iron concentration data are shown in Figure S6.

## Discussion

### Substrate-dependent fluoride inhibition in pure cultures

All six typical sulfur- and iron-oxidizing bioleaching bacteria tested in this study displayed higher tolerance to fluoride when grown on ferrous iron compared to sulfur as substrate. This was observed particularly for *At. ferrooxidans*. While no activity was detected at 0.75 mM F using sulfur as a substrate, *At. ferrooxidans* was still active at 1 mM F when grown on ferrous iron. The same was observed for *S. thermosulfidooxidans*, displaying no activity when fluoride was added to sulfur-grown cultures, but was still active at 1.5 mM F when grown on Fe^2+^. These results suggest that fluoride toxicity is influenced by the type of substrate, consistent with findings in the literature(Brierley & Kuhn 2010; Sicupira et al. 2011; Ma et al. 2017). As previously described, fluoride does not necessarily lead to cell death; it can also lead to increased ATP consumption for an increased out-pumping of protons. Consequently, the cells require more energy and thus substrate, which leads to a reduction in biomass production (Veloso et al. 2012), reduced microbial activity and sulfuric acid formation occurs. It should however be noted that the selected acidophiles have different growth optima (see Table 1). The optimum for *At. thiooxidans* is pH 2.0–3.0, but it can grow within a range of pH 0.5–5.5 (Kelly & Wood 2000). *At. ferrooxidans* is only active at a pH range of 1.3–4.5 and has its optimum at approximately pH 2.5 (Kelly & Wood 2000). The growth conditions applied in this study, especially a starting pH of 1.8–1.6, are therefore more suited for *At. thiooxidans*. Also, the starting pH of the medium is close to the pH limit of 1.3 for *At. ferrooxidans*, which might also cause inhibition due to low pH. For better comparison with the mixed culture studies, cultivation of *At. caldus* was carried out at 42 °C, while its optimum is 45 °C (Kelly & Wood 2000). *S. thermosulfidooxidans* has been reported to grow between pH 1.5 and 5.0 with an optimum around pH 3.0 (Zhang et al. 2021). Thus, a pH of 1.8 at the beginning of the experiment is already at the lower limit for its microbial activity as also indicated by the fact that there is no further drop below pH 1.4 in the experiments with additional fluoride stress.

Considering the inoculum cell counts from pre-cultures grown on sulfur under the experimental conditions without fluoride, *At. thiooxidans* already showed one of the highest cell densities compared to *S. thermosulfidooxidans* and *At. ferrooxidans*. Interestingly, *At. caldus* was also inoculated at high density but exhibited lower fluoride tolerance. For the pre-cultures of the iron-oxidizing bacteria, very similar inoculum cell densities were observed, with *S. thermosulfidooxidans* and *L. ferriphilum* showing the lowest values, presumably due to less optimal cultivation conditions. These observations also indicate that both the initial cell density and growth conditions (pH and temperature) influence the microbial activity under fluoride stress.

The pH plays a crucial role in determining the HF concentration, as it is substantially lower in sulfur-oxidizing cultures compared to iron-oxidizing cultures since microbial iron oxidation is a proton-consuming reaction (Johnson & Hallberg 2009). As described in literature, the concentration of free fluoride increases with rising pH (Brierley & Kuhn 2010; Li et al. 2019). Li et al. (2019) showed that at pH 5.0, more than 90% of the fluoride is present as F^−^. At pH < 2.0, more than 95% of the fluoride is present as HF (Li et al. 2019) and at pH 1.5 fluoride should be almost completely present as HF (Denham & Millings 2003). Therefore, pH dictates the HF concentration in the medium (Eq. 8) and whether excess protons additionally restrict microbial activity. The concentration of HF can be deduced from the concentration of F^−^ as shown in Eqs. (8–10) (Li et al. 2019):

8 $$\mathrm H^+\;+\mathrm F^-\;\rightarrow\mathrm{HF}$$

9 $${\mathrm{pK}}_a=-\mathrm log\frac{\left[H^+\right]\lbrack F^-\rbrack}{\lbrack HF\rbrack}$$

10 $$\mathrm{pH}=\mathrm{pKa}+\mathrm log\frac{\lbrack F^-\rbrack}{\lbrack HF\rbrack}$$

When using ferrous iron as a substrate during these studies, the final pH was 2.0–2.4 (data not shown), which is higher compared to the experiments with sulfur as substrate and a final pH ≤ 1.3. Therefore, a lower HF concentration can be expected for the iron-grown cultures. As shown by Ma et al. (2017), more than 85% and 80% of the fluoride was present in the form of HF at pH 2.0 and 2.2, respectively. When ferric iron at a Fe^3+^:F ratio of 10:1 was added to the system, the HF concentration was less than 2% of the total fluoride (Ma et al. 2017).

Ma et al. (2013) also investigated the growth of various sulfur-grown acidophiles at different fluoride concentrations, with fluoride added only during the exponential growth phase. *At. thiooxidans* showed decreased cell counts at 2.4 mM F. *At. caldus* showed only a slight increase in cell counts at 2.4 mM F and no clear drop in the pH after fluoride addition (Ma et al. 2013). Even though the study by Ma et al. (2013) reported considerably higher fluoride concentrations, it should be noted that they added the fluoride during the exponential growth phase. During this phase, attachment of bacteria to the sulfur particles and activation of the sulfur and oxidation had already started, which potentially allows tolerance towards higher fluoride concentrations. Similar to the data obtained in this study, *At. thiooxidans* appears to tolerate higher fluoride concentrations than *At. caldus*. Ma et al. (2017) also investigated the inhibition of fluoride added to sulfur-grown *At. ferrooxidans*. A slight drop in pH and an increase in cell counts were observed when 0.1 mM fluoride was added in the lag phase, while above 0.25 mM F, complete inhibition was described (Ma et al. 2017), which is higher than the inhibitory concentration observed for the sulfur-grown *At. ferrooxidans* culture tested in this study. The pH value, which is substantially lower during microbial sulfur oxidation than during iron oxidation, also has a major influence on the fluoride tolerance as the concentration of HF in the medium is determined by the pH. In addition, the enhanced fluoride tolerance of ferrous iron-grown cultures can be explained by the fact that ferric iron can also complex fluoride to, e.g., form FeF^2+^ and thereby reduce the concentration of HF in the medium (Rodrigues et al. 2015, 2019; Ma et al. 2017). The bacteria are capable of reducing the concentration of HF ions in the medium independently through microbial iron oxidation and ferric iron production (Ma et al. 2017). Among the iron-oxidizing bacteria tested, 0.5 mM F does not appear to have any notable effect on the microbial iron oxidation activity. However, it should be noted that the precultures were already cultivated on ferrous iron, which means that ferric iron was also transferred during inoculation, which affects the HF concentration. Ma et al. (2013) also investigated the influence of fluoride added during the exponential growth phase on the iron oxidation of various acidophilic bacteria. *At. ferrooxidans* was able to grow even at concentrations of 12 mM F after a decline in cell number. At 2.4 mM F, there was only a slight reduction in the number of cells compared to the positive control. *L. ferriphilum* only showed a clear increase in cell number at 2.4 and 4.8 mM F after a short stagnation, while at higher concentrations a decrease in the cell count was observed. For *S. thermosulfidooxidans*, no increase in cell count after fluoride addition was observed, but iron oxidation was still present at 2.4 mM F (Ma et al. 2013). This difference could be explained by the fact that yeast extract was added during the cultivation of *S. thermosulfidooxidans*. *Sulfobacillus* species are capable of assimilating organic and inorganic forms of carbon. They can derive energy from the oxidation of ferrous iron and various reduced sulfur species. They require suitable carbon compounds, such as yeast extract, for continued growth (Norris et al. 1980). It is therefore assumed that the carbon for biomass production is not exclusively derived from CO_2_ (Shiers et al. 2010). Therefore, yeast extract was also added in these experiments for the cultivation of *S. thermosulfidooxidans*. However, since this mixotrophic species can also metabolize organic substrates, the question arises as to whether there is a possible preference for a substrate under stress conditions. Shiers et al. (2010) observed a uniform substrate utilization by iron-adapted *S. thermosulfidooxidans*, which preferred the oxidation of ferrous iron over tetrathionate, while the tetrathionate-adapted cells oxidized both substrates concurrently. However, it is well known that adaptation of microorganisms to a certain substrate affects their growth behavior.

Compared to the studies carried out here, a higher fluoride tolerance was determined for the respective strains by Ma et al. (2013) for iron-grown cultures. It should, however, be noted that the authors added fluoride during the exponential growth phase rather than before inoculation. Furthermore, the medium used may also play a role. While HBS-minimal salt medium was used in this study, 9 K medium with 44.7 g/l FeSO_4_·7H_2_O (160.8 mM Fe^2+^) was used by Ma et al. (2013). Accordingly, a certain concentration of ferric iron was present at the time of inoculation, affecting the concentration of HF in the medium and therefore increasing the fluoride tolerance. Veloso et al. (2012) also described delayed iron oxidation for *S. thermosulfidooxidans* when 0.25 mM F was added during the lag phase. While our study could not confirm the results reported by Ma et al. (2013) and Veloso et al. (2012), the low pH of 1.5 used in these studies compared to our set up at pH 1.8 might have already had an inhibitory effect. In addition, shorter cultivation times (50–60 h) were used by the authors, which makes a comparison difficult. Our studies showed that *S. thermosulfidooxidans* requires at least four days until iron oxidation can be measured, compared to *At. ferrooxidans*, for example, showing this after only two days. Ma et al. (2017) also reported that the iron oxidation of *Af. ferrooxidans* was not affected by 0.25 mM F, while from 0.5 mM to 1.25 mM F the activity decreased with increasing fluoride concentration. At 1.5 mM F, no microbial iron oxidation was observed during the cultivation period tested. The results reported by Ma et al. (2017) for *At. ferrooxidans*, *L. ferrooxidans*, and *L. ferriphilum* are similar to the data reported in this study, also showing no microbial activity at 1.5 mM F (added before inoculation). However, when fluoride was added during the exponential growth phase, the bacteria showed much higher fluoride tolerance. The microbial iron oxidation also remained at 2.5 mM F, whereby a slight delay in iron oxidation was observed at 5.0 mM F or higher. Nevertheless, iron oxidation was not greatly inhibited even at 10.0 mM F (Ma et al. 2017).

### Substrate-dependent fluoride inhibition in enrichment and mixed cultures

The positive effect of ferric iron was also confirmed by the results of the cultivation tests with the enrichment culture, which showed enhanced sulfur oxidation activity in the presence of ferric iron. Remarkably, sulfur oxidation activity was still detectable at fluoride concentrations of up to 8 mM F. However, as the enrichment culture still exhibits a slight activity even at 4 mM fluoride (without the addition of iron), it can be assumed that they tolerate higher fluoride concentrations than the other acidophilic sulfur-oxidizing bacteria. Adaptation of acidophilic microorganisms to higher fluoride concentrations was already described in the literature (Wang & Qiu 2011; Qian et al. 2013; Zhou et al. 2019). Wang & Qiu (2011), for example, were able to grow them at 45 mM F^−^ after continuous adaptation (Wang & Qiu 2011). A strain of *At. ferrooxidans* grew in the presence of 2.1 mM F after long-term adaptation (Qian et al. 2013). Therefore, the fluoride tolerance of the enrichment culture might also increase with time.

Monitoring of the moderately thermophilic mixed culture revealed that sulfur oxidation activity was present even at higher fluoride concentrations, in contrast to the tested pure cultures. However, microbial iron oxidation in the mixed culture was not observed at fluoride concentrations above 0.5 mM F. In comparison, the mesophilic mixed culture-maintained sulfur oxidation activity up to 1.0 mM F and iron oxidation up to 1.5 mM F, indicating a lower inhibitory effect of fluoride than in the respective pure cultures. This result differs from the moderately thermophilic culture. Whereas the pH of the pure cultures of iron-oxidizers was > 2.0, the pH of the moderately thermophilic mixed culture was already 1.4 (1.0 mM F) or 1.6 (1.5 mM F) after 2 days and pH 1.0 (1.0 mM F) or 1.2 (1.5 mM F) after 4 days. Due to the low pH, the HF and H^+^ concentration was higher in the mixed cultures compared to the pure cultures. Based on the optimum growth conditions of the bacteria (Table 1), it becomes apparent that *S. thermosulfidooxidans* should no longer be very active at a pH < 1.5. *L. ferriphilum* grows optimally at pH as low as 1.4, with its activity decreasing below this pH value. *At. caldus*, on the other hand, is still active at a pH as low as 1.0. Also, *At. caldus* has been reported to contribute to the reduction of ferric iron at lower pH (Malik & Hedrich 2022), which might have an effect on the redox potential.

It should also be noted that in mixed cultures, the concentrations of individual species are typically lower than those in pure culture experiments. This difference can substantially influence microbial activity, such as oxidation rates. The moderately thermophilic mixed culture comprises three species, one of which is capable of dual oxidation. The lower abundance of iron-oxidizing bacteria may contribute to the reduced iron oxidation observed in the mixed culture compared to pure cultures. In contrast, the mesophilic mixed culture consists of only two species, with both species not dedicated to sole sulfur oxidation. Consequently, inoculation likely involved higher cell numbers per species. *L. ferrooxidans* and *At. ferrooxidans* both oxidize iron, which may also explain the improved tolerance of the culture, as evidenced by enhanced Fe^3^⁺ production and an increase in pH relative to the moderately thermophilic mixed culture. Thus, the structure of the microbial community plays a crucial role in overall performance. In future studies, community analyses would be valuable for gaining further insights into fluoride inhibition.

Li et al. (2015) investigated fluoride inhibition on a mesophilic and moderate thermophilic mixed culture of *At. ferrooxidans*, *L. ferriphilum*, *S. thermosulfidooxidans*, *At. thiooxidans*, and *At. caldus* (at 40 °C). The iron oxidation rates were clearly inhibited, while sulfur oxidation was affected less. The authors examined the microbial community dynamics, whereby *S. thermosulfidooxidans* was most inhibited. Therefore, it is also possible that in the experiments described in this study, *S. thermosulfidooxidans* was also inhibited. The pH-dependent fluoride tolerance was investigated for iron-grown *At. ferrooxidans* and *L. ferriphilum* at pH between 1.5 and 4.0 and fluoride concentrations of 10–40 mg/l (Li et al. 2019). Slow bacterial growth was observed at pH 2.0 and 0.53 mM F, pH 3.0 and 1.05 mM F, and pH 4.0 and 2.11 mM F, whereas no bacterial growth was observed at pH 1.5 and 0.53 mM F, pH 2.0 and 1.05 mM F, and pH 3.0 and 2.11 mM F. Finally, the study demonstrated that the mechanism of fluoride toxicity is mainly affected by pH (Li et al. 2019). In this study, the mesophilic mixed culture shows activity at higher fluoride concentrations, with the pH only reaching approx. 1.5 on day seven. In comparison, the moderately thermophilic mixed culture displayed a pH far below 1.5 during cultivation and thus lower iron oxidation activity. The data also clearly show that the pH value has a major effect on microbial activity. The results demonstrate that the use of mixed cultures can enhance fluoride tolerance, particularly for sulfur oxidation. This is particularly relevant since mixed cultures are commonly used in bioleaching processes (Hedrich et al. 2016; Vera et al. 2022). Improved fluoride tolerance was also observed for the iron oxidizers in the mesophilic mixed culture. However, further studies with additional microbial consortia are needed to confirm these findings. For example, the addition of the sulfur-oxidizing *At. thiooxidans* to the mesophilic mixed culture could further decrease the pH, allowing conditions more comparable to those observed in the moderately thermophilic culture. These findings also highlight the strong influence of pH on fluoride inhibition in mixed culture systems.

In summary, the study confirms the substrate dependence of the fluoride tolerance in acidophilic bacteria, as already described by Ma et al. (2017). The composition of the growth medium, especially with respect to the presence of ferric iron, plays a substantial role. Furthermore, a species-dependent fluoride tolerance is indicated; however, it could not be confirmed as cultivation was not carried out under optimum conditions for each strain. The growth phase at which the bacteria are exposed to fluoride also has an influence on fluoride resistance, as fluoride tolerance is lower when added before inoculation compared to addition during exponential growth when ferric iron is already present or sulfur has been activated. The results also indicate the major influence of the pH of the medium. It would therefore be useful to compare it iron oxidation at different pH values in order to better evaluate the effect of the pH on microbial iron oxidation and to compare with sulfur oxidation. With regard to bioleaching applications, even low fluoride concentrations have been shown to cause inhibitory effects, which limits the efficiency of bioleaching processes. In addition, iron oxidation can positively influence the activity of bioleaching microorganisms. However, a challenge is the low pH typically required for the activity of acidophilic microorganisms.

### Complexation of fluoride by ferric iron or aluminum

The use of aluminum to complex fluoride during bioleaching of fluoride-containing ores has already been described in the literature (Sicupira et al.; 2011; Veloso et al. 2012). Complexation by ferric iron may also be a viable approach (Rodrigues et al. 2016, 2019; Ma et al. 2017), as Fe^3^⁺ is often present in bioleaching systems. In this study, these strategies were investigated in more detail to gain a better understanding of fluoride behavior during bioleaching of fluoride-containing materials and to support future process optimization. Ma et al. (2017) described a linear relationship between HF concentration and the increase in NaF and ferric iron concentrations. For example, a concentration of 10.0 mM NaF and an Fe/F ratio of 10:1 resulted in a very low HF concentration (Ma et al. 2017). As already shown in the previous experiments, the presence of ferric iron has an influence on the HF concentration besides the pH of the medium. The ferric iron concentration needed to counteract fluoride inhibition was determined using sulfur- and iron-grown cultures of *At. thiooxidans*, *At. ferrooxidans*, and *L. ferrooxidans*. While for *At. thiooxidans* a F:Fe^3+^ ratio of 1:7.5 was already sufficient to achieve reasonable activity under these cultivation conditions, *At. ferrooxidans* required a higher ratio of at least 1:10 when grown on sulfur. This difference may be related to the previously discussed variations in optimal growth conditions. Taking cell counts into account, *At. thiooxidans* also exhibited the highest inoculum cell count compared to *At. ferrooxidans* on sulfur, which may contribute to the observed difference in the optimal ratio. Compared to *L. ferrooxidans*, *At. ferrooxidans* seems to require a lower F:Fe^3+^ ratio of 1:7.5 for high iron oxidation activity. Initial experiments already showed that *At. ferrooxidans* exhibited a slight increase in iron oxidation activity from day two when 1 mM F was added, whereas *L. ferrooxidans* only showed this from day four (Fig. 3). Thus, a F:Fe^3+^ ratio of 1:10 could be sufficient for *L. ferrooxidans*. Ma et al. (2019) described an approach to overcome fluoride inhibition by stepwise addition of ferric iron, thereby enabling the bioleaching of chalcopyrite using *At. ferrooxidans*. The applied F:Fe^3+^ ratio of 1:6 for chalcopyrite bioleaching closely corresponds to the ratio of 1:7.5 determined in this study. Furthermore, the data presented here are consistent with the findings of Ma et al. (2017), who reported that an Fe:F^3+^ ratio of 10:1 was effective in minimizing fluoride toxicity. Later, they also described that a minimum molar ratio of 1:3 F:Fe^3+^ reduced the detrimental influence of fluoride on microorganisms (Ma et al. 2019).

In addition to ferric iron, aluminum has been reported as an effective complexing agent to mitigate fluoride inhibition (Brierley & Kuhn 2010; Sicupira et al. 2011; Veloso et al. 2012). Accordingly, experiments with *At. thiooxidans* and a mixed culture of *S. thermosulfidooxidans*, *L. ferriphilum*, and *At. caldus* were conducted to identify the optimal Al:F ratio for growth. The results of both cultures showed a difference in the required Al:F ratio. For sulfur-grown *At. thiooxidans*, a ratio of 1.5:1 is required, while a ratio of 1:1 is sufficient when ferric iron is present in the medium for microbial activity. The moderately thermophilic mixed culture grown on sulfur and ferrous iron required an Al:F ratio of 2:1 for high iron and sulfur oxidation activity. While the ferric iron present in the *At. thiooxidans* cultures also complexes fluoride from the beginning, in the moderately thermophilic mixed culture, the ferrous iron requires oxidation to also counteract with the fluoride ions. This can generally present a challenge during the bioleaching of fluoride-containing minerals or residues. During bioleaching, iron is released from the ore in its ferrous form, and fluoride is simultaneously mobilized. Even at low concentrations, the presence of fluoride can inhibit bacterial growth before the ferrous iron is oxidized by microbial activity. This inhibitory effect can be mitigated by the addition of aluminum to the leaching solution, which complexes dissolved fluoride ions (Veloso et al. 2012; Rodrigues et al. 2019).In the literature, Al:F ratios ranging from 1.25:1 (Rodrigues et al. 2016) to 2:1 (Veloso et al. 2012) have been reported. As described in Rodrigues et al. (2016; 2019), the dissolved fluoride concentration is determined by the Al and ferric iron concentration (Eq. 11). The mass ratio between the total fluoride, total aluminum, and total ferric iron concentrations shows that a low value indicates the presence of the main elements for fluoride complexation (Rodrigues et al. 2016).

11 $$\frac{\left[F\right]tot}{\left[Al\right]tot+\left[Fe^{3+}\right]tot}$$

A higher resulting value indicates lower concentrations of aluminum and ferric iron concentrations present in the medium, which could reduce the HF concentration. It should be noted that the required concentrations of Al and ferric iron ions differ for the complexation of the fluoride, as a lower Al:F ratio is required compared to the F:Fe^3+^ ratio. This fact can be explained, for example, by directly comparing data for *At. thiooxidans* in this study. While a F:Fe^3+^ ratio of 1:7.5 was necessary, it only required an Al:F ratio of 1.5:1 (without the addition of ferric iron). Of all common ligands, F^−^ binds more strongly to Al^3+^ than to Fe^3+^ (Martin 1996) since the aluminum-fluoride complexes have higher stability constants, e.g., logK_1_ = 6.13, logK_2_ = 5.02, and logK_3_ = 3.85 (at 25 °C) (Goldstein 1964). In addition, the rates of reactions k₁ = 20.7 M^−1^ s^−1^ at 25 °C and k_2_ = 471 M^−1^ s^−1^ when HF is in the system have been described (Nemes et al. 1998). For ferric fluoride complexes, the stability constants are lower, with logK_1_ = 5.3, logK_2_ = 4.46, and logK_3_ = 3.22 (Goldstein 1964). In addition, rates of k_1_ of approx. 40 M^−1^ s^−1^ and k_2_ = 11 M^−1^ s^−1^ at 25 °C are described (Hudis & Wahl 1953). Thus, aluminum reacts much faster and forms thermodynamically more stable fluoride complexes than ferric iron. Subsequently, to reduce the HF concentration in the medium as well as to minimize or eliminate the inhibitory effect, more ferric iron is needed to achieve the same effect as with aluminum. The aluminum complexation appears to be the more effective approach for bioleaching applications compared to ferric iron in order to overcome inhibition of the microorganisms. Although inhibitory effects by aluminum on the activity of bioleaching bacteria have been reported, this was only effective at concentrations > 10 g/l Al (Veloso et al. 2012; Sicupira et al. 2011). Previous studies also demonstrated that the complexation reactions are temperature dependent, with reaction rates increasing at higher temperatures (Hudis & Wahl 1953; Plankey et al. 1986; Nemes et al. 1998). It is therefore possible that complexation occurred more rapidly at 42 °C than at 30 °C. However, this assumption requires validation through comparative experiments. Nevertheless, this aspect should be considered in the application and planning of bioleaching processes.

While ferric iron can contribute positively, even low fluoride concentrations were shown to inhibit microbial activity. Thus, the addition of aluminum offers a more reliable strategy for bioleaching performance in fluoride-containing systems. The final molar ratio of aluminum and fluoride is determined by various factors, such as pH, ferric iron concentration, and dissolution rate of fluoride, e.g., during bioleaching of fluoride-containing minerals.

## Conclusion

During bioleaching of fluoride-containing minerals or residues, fluoride is present as HF due to the low pH, which can inhibit microbial activity. In this study, fluoride inhibition of typical acidophilic iron- and sulfur-oxidizing bacteria was investigated. It has been shown that using sulfur as a substrate, the maximum fluoride concentration that *At. thiooxidans* tolerates is 0.5 mM F, whereby *S. thermosulfidooxidans* displayed microbial iron oxidation even at 1.5 mM F. In mesophilic mixed cultures, microbial iron and sulfur oxidation occurred even at elevated fluoride concentrations compared to the pure cultures, whereas in moderately thermophilic mixed culture, only sulfur oxidation was confirmed at higher fluoride concentrations. Fluoride tolerance is mainly dictated by pH, as the HF concentration is pH dependent and increases sharply with decreasing pH. In addition to the pH value, the presence of ferric iron determines the HF concentration, as it complexes the fluoride ions. More detailed investigations on ferric iron and fluoride complexation showed that a ratio of 1:7.5 is already sufficient for active sulfur oxidation by *At. thiooxidans*, while for the iron-oxidizing *L. ferrooxidans*, a ratio of 1:10 is required to achieve microbial activity. Besides the effect of ferric iron, complexation of fluoride by aluminum achieved promising results, showing that for a moderately thermophilic mixed culture of iron- and sulfur-oxidizing acidophiles, an Al:F ratio of 2:1 was required, while for sulfur-grown *At. thiooxidans*, a lower ratio of 1.5:1 was sufficient for high microbial activity.

In summary, this study shows that the inhibition of bacteria by fluoride is primarily determined by the pH value, but also influenced by substrates used. Furthermore, fluoride inhibition is reduced by the presence of ferric iron and can be minimized by adding aluminum. This study provides insights into the toxic effects of fluoride during bioleaching of fluoride-containing materials and strategies to avoid inhibition of the microbial activity.

## Supplementary Information

Below is the link to the electronic supplementary material.

ESM 1 (DOCX 1.94 MB)

## Data Availability

All data generated or analyzed during this study are included in this published article. Additional data are available from the corresponding author upon reasonable request.
