# Supplementary material for: Fluoride toxicity and mitigation strategies in acidophilic bioleaching microorganisms
Source: Appl Microbiol Biotechnol. 2026 Jan 23;110:32. doi: 10.1007/s00253-025-13677-x (PMC12831704; doi:10.1007/s00253-025-13677-x)
Supplement: Supplementary file 1 [file 253_2025_13677_MOESM1_ESM.docx]

**Fluoride Toxicity and Mitigation Strategies in Acidophilic Bioleaching Microorganisms**

Mareike Thea Fritze^1^, Sabrina Hedrich^1^

^1^Institute of Biosciences, TU Bergakademie Freiberg, Leipziger Str. 29, 09599 Freiberg, Germany

[mareike-thea.fritze@bio.tu-freiberg.de](mailto:mareike-thea.fritze@bio.tu-freiberg.de), ORCID: 0009-0007-2953-2464
[sabrina.hedrich@bio.tu-freiberg.de](mailto:sabrina.hedrich@bio.tu-freiberg.de), ORCID: 0000-0001-6125-5566

**Fig. S1:** Monitoring of ferrous iron concentration in pure cultures of iron-oxidizing bacteria with different fluoride concentrations; *L. ferrooxidans* (a), *At. ferrooxidans* (b), *S. thermosulfidooxidans* + 0.02 % yeast extract (c) and *L. ferriphilum* (d), PC = positive control without addition of fluoride. (Data represent mean values of triplicate set ups with standard deviation)

**Fig. S2:** Monitoring of ferrous iron concentration in mixed cultures of iron-and sulfur oxidizers under addition of different fluoride concentrations; moderately thermophilic culture of *S. thermosulfidooxidans*, *L. ferriphilum* & *At. caldus* (a, b); mesophilic culture of *L. ferrooxidans* & *At. ferrooxidans* (c, d), PC = positive control without addition of fluoride. (Data represent mean values of triplicate set ups with standard deviation)

**Fig. S3:** Monitoring of changes in redox potential (vs. Ag/AgCl) and ferric iron concentration of the enrichment culture at different fluoride concentrations when grown on Fe^3+^ and sulfur, PC = positive control without addition of fluoride. (Data represent mean values of triplicate set ups with standard deviation)

**Fig. S4:** Monitoring of ferrous iron concentration in pure cultures of iron-oxidizing bacteria at different molar ratios of fluoride and Fe^3+^ (F: Fe^3+^), *At. ferrooxidans* (a), *L. ferrooxidans* (b) on Fe^2+^ , CC= chemical control,
PC = positive control without fluoride. (Data represent mean values of triplicate set ups with standard deviation)

**Fig. S5*:*** Monitoring of ferrous iron concentration in pure cultures of *At. thiooxidans* at different molar ratios of aluminum and fluoride (Al:F). *At. thiooxidans* grown on 1% (w/v) sulfur (a), *At. thiooxidans* grown on sulfur 1% (w/v) and 30 mM Fe^3+^ (b, c); CC= chemical control, PC = positive control without addition of fluoride. (Data represent mean values of triplicate set ups with standard deviation)

**Fig. S6*:*** Monitoring of ferrous iron concentration of a moderate thermophilic mixed culture
(*S. thermosulfidooxidans*, *L. ferriphilum* & *At. caldus*) grown on 1% sulfur (w/v) and 50mM Fe^2+^ at different molar ratios of Al:F, CC= chemical control, PC = positive control without addition of fluoride. (Data represent mean values of triplicate set ups with standard deviation)
